# Efficacy and safety of icotinib in treating non-small cell lung cancer: a systematic evaluation and meta-analysis based on 15 studies

**DOI:** 10.18632/oncotarget.13509

**Published:** 2016-11-22

**Authors:** Rong Biaoxue, Liu Hua, Gao Wenlong, Yang Shuanying

**Affiliations:** ^1^ Department of Respiratory Medicine, First Affiliated Hospital, Xi’an Medical University, Xi’an, China; ^2^ Department of Respiratory Medicine, Gansu Provincial Hospital, Lanzhou, China; ^3^ Department of Statistics and Epidemiology, Medical College, Lanzhou University, Lanzhou, China; ^4^ Department of Respiratory Medicine, Second Affiliated Hospital, Xi’an Jiaotong University, Xi’an, China

**Keywords:** icotinib, non-small cell lung cancer, NSCLC, meta-analysis, efficacy

## Abstract

Icotinib is a new epidermal growth factor receptor (EGFR) tyrosine kinase inhibitor (TKI) that developed and used in China; this work was to evaluate its efficacy and safety in treating non-small cell lung cancer (NSCLC). Clinical studies evaluating the efficacy and safety of icotinib in treating NSCLC were identified from the databases of Medline, Web of Science, Embase and Cochrance Library. Pooled efficacy and safety of icotinib were calculated through a series of predefined search strategies. A total of 15 studies with 2,304 patients were involved in this study. The overall response rate (ORR) and disease control rate (DCR) of icotinib were 40.99% (95% CI: 33.77% to 48.22%) and 77.16% (95% CI: 51.43% to 82.31%). The pooled progression-free survival (PFS) and overall survival (OS) were 7.34 months (95% CI: 5.60 to 9.07) and 14.98 months (95% CI: 9.78 to 20.18). Patients with EGFR mutations exhibited better ORR (OR = 3.67, *p* < 0.001), DCR (OR = 1.39, *p* = 0.001) and PFS (11.0 ± 0.76 vs. 1.97 ± 0.82 months). Moreover, patients with rash had a higher ORR (OR = 2.14, *p* = 0.001) than those without rash. The common adverse effects (AEs) included skin rash (31.4%), diarrhea (14.2%), pruritus (6.7%) and hepatic toxicity (3.8%) and most of them were well tolerated. In conclusion, Icotinib is an effective and well tolerated regimen for Chinese patients with advanced NSCLC. Further randomized trials with large population are required to provide stronger evidence for icotinib in treating NSCLC.

## INTRODUCTION

With the increasing environmental pollution and food safety problems, lung cancer in the People's Republic of China is going up continuously [1]. It is estimated that there will be about 733,000 newly diagnosed invasive lung cancer cases in 2015 in China, and about 610,000 Chinese will die from lung cancer in 2015 [2]. Although studies on the early diagnosis and individual treatment of lung cancer have made great progress, NSCLC is still a very malignant disease characterized by a high incidence of mortality. Now, rapid advances in genomics and proteomicss of lung cancer have shown that targeted therapy against to specific gene and protein is more effective in treating advanced NSCLC such as tyrosine kinase inhibitors (TKIs) which targets mutated epithelial growth factor receptors (EGFRs) [3]. Research shows that the EGFR mutations is crucial because TKIs seem to be more effective in NSCLC with EGFR mutations [4]. Many trials suggest that TKIs treatment in patients with EGFR-mutated NSCLC has a significant survival benefit, and other combined treatment of chemotherapy and TKIs also significantly improve progression-free survival (PFS) in advanced NSCLC [5].

Icotinib has been approved by the China Food and Drug Administration (CFDA) as an oral EGFR TKI in June 2011 [6], which was first developed by Zhejiang Bata Pharma Ltd (Hangzhou, Zhejiang, China, Patent No.WO2003082830) [7]. Its chemical structure is 4-((3-ethynylphenyl) amino)-6, 7-benzo-12- crown-4- quinazoline hydrochloride and structural formula is C22H21N3O4·HCl, and molecular weight is 427.88 [8]. Preclinical and clinical researches have demonstrated that icotinib has a better efficacy in treating advanced NSCLC [7, 9], which specifically and competitively bind to tyrosine kinase, inhibit the enzymatic activity of EGFR, block the related signal conduction and thereby reduce cancer cell growth [10]. Here, we summed up the Phase I, II, and III clinical trials of icotinib and discussed its current clinical application and future research directions.

## RESULTS

### Literature identification

**Figure 1:**
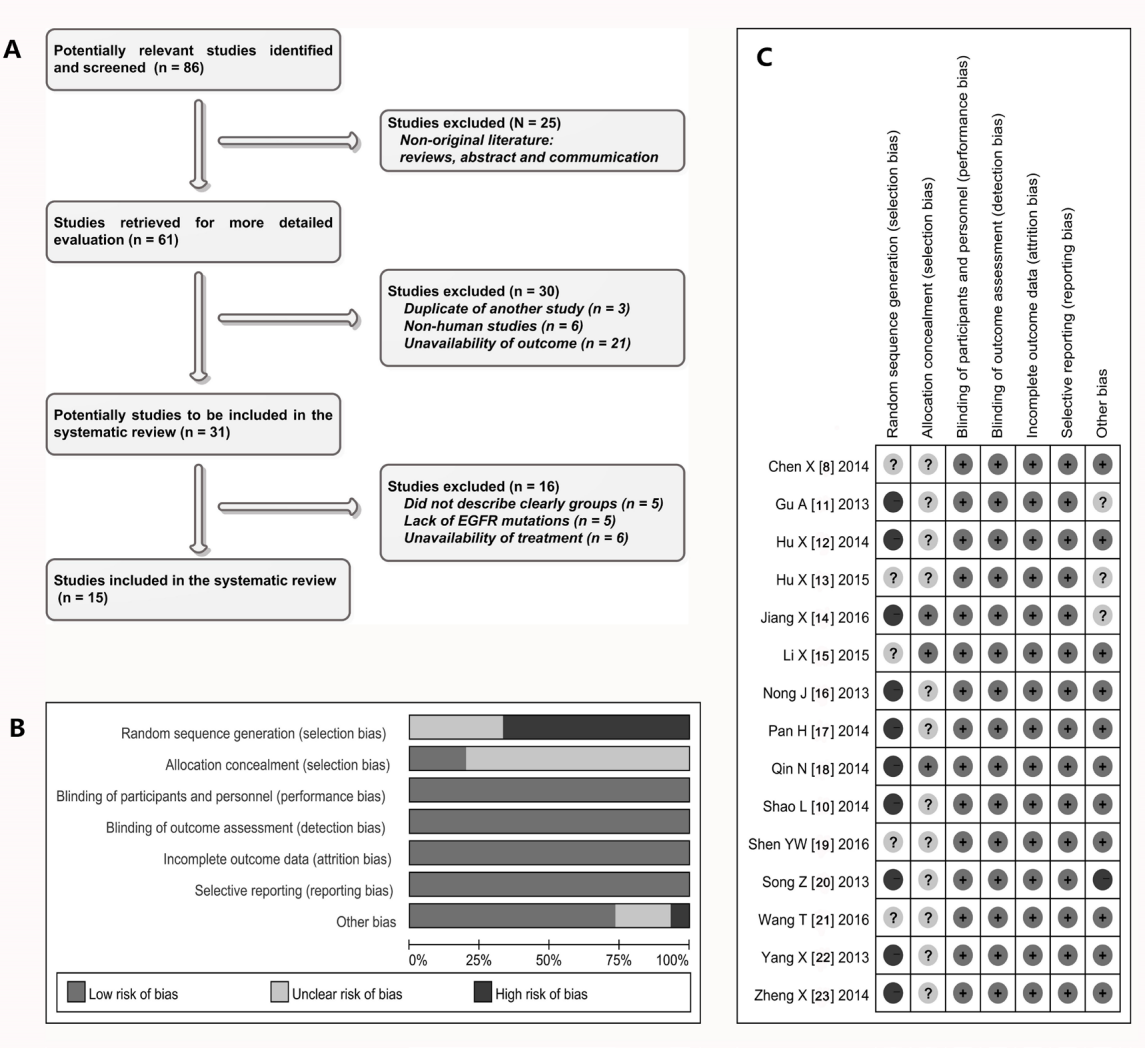
Selection and assessment of literature (**A**) Studies were retrieved from the electronic bibliographic databases such as PubMed, Embase, Cochrane Library and SCI database; (**B**–**C**) according to the criteria made by the Cochrane Handbook (Version 5.0.1), the random assignment of studies was not satisfactory, but there are relatively few other methodological problems in those studies.

As shown in Figure 1A, preliminary searches defined 86 relative studies that described the relevant investigations on icotinib and NSCLC. Of them, we firstly excluded 25 studies because they were not original research documents. Of the 61 publications that remained, we abandoned 30 studies once more because of data duplication and unavailability of outcome. Of the remained 31 studies, we finally abolished other 16 studies due to the following reasons: unclear groups; lack of data on EGFR mutations; and some received other complicated treatment simultaneously. At the end of the identification process, 15 studies [8, 10–23] were considered eligible for further analysis, which included a total of 2,304 individuals (Table 1).

**Table 1:** Clinical characteristics of eligible studies

Authors	Year	Country	All cases (N)	Gender (N)		Ages		Histology (N)		Tumor stage (N)		EGFR mutation	
				Male	Female	Male	Female	LAC	Others	I-II	III-IV	Positive	Negative
Song Z [20]	2013	China	49	26	23	57		44	5	−	49	36	13
Gu A [11]	2013	China	89	49	40	−		75	14	0	89	4	2
Yang X [22]	2013	China	56	21	35	67.88		−	−	−	56	18	2
Nong J [16]	2013	China	60	26	34	58		54	6	4	56	23	9
Shao L [10]	2014	China	149	102	47	60 (34–84)		103	46	−	149	12	12
Qin N [18]	2014	China	101	45	56	62 (35–86)		91	10	0	101	35	11
Zheng X [23]	2014	China	42	7	35	62.5 (35−85)		42	−	2	40	2	6
Hu X [12]	2014	China	1026	480	546	63 (21−95)		774	252	43	983	665	214
Pan H [17]	2014	China	69	38	31	56		61	8	−	69	51	18
Chen X [8]	2014	China	82	41	41	64		75	7	0	82	19	63
Li X [15]	2015	China	124	55	69	59.5 (36−80)		115	9	−	124	99	25
Hu X [13]	2015	China	124	65	59	57 (30−73)		93	30	−	124	51	−
Wang T [21]	2016	China	67	28	39	59		64	3	57	10	38	29
Shen YW [19]	2016	China	35	19	16	63		35	−	0	35	35	0
Jiang X [14]	2016	China	231	106	125	57 (31−85)		221	10	0	231	231	0

Abbreviations: N, cases; LAC, lung adenocarcinoma; EGFR, epidermal growth factor receptor.

### Description of literature

As can be seen from the Table 1, 15 studies [8, 10–23] investigated the efficacy and safety of icotinib in treating NSCLC from 2013 to 2016, including 1108 men and 1196 women with a mean age of 60.42 years. All patients were Chinese and the lung adenocarcinomas accounted for 92% of all patients (1847/2304). Among all patients, 2198 were diagnosed at III-IV of clinical stage, which accounted for 95.4% of patients (2198/2304). As shown in Table 2, 13 studies [8, 10, 11, 15–23] were single central but two studies were multiple center [12, 13]. Fourteen of 15 studies showed relevant data of EGFR mutations [8, 10–12, 14–23], in which patients with EGFR mutations accounted for 75.8% (1268/1672) and those without mutations accounted for 24.3% (404/1672). Twelve studies reported the detailed follow-up data [8, 10, 13–21, 23] and six studies showed the methods of testing for EGFR mutations [15, 18–20, 22, 23]. All studies described the methods of administration in detail and finished the efficacy evaluation.

**Table 2:** Methodology and quality of inclined studies

Authors	Study design	Region	Test method	Follow-up	Missing data	Therapy	Administration	Drug time	TREND
Song Z [20]	Retrospective	Single center	Pyrosequencing	49	0	FL and MT	125 mg; 3/day	DP and IT	51
Gu A [11]	Retrospective	Single center	−	−	−	FL and MT	125 mg; 3/day	DP and IT	48
Yang X [22]	Retrospective	Single center	MELA	−	−	FL	125 mg; 3/day	DP and IT	55
Nong J [16]	Retrospective	Single center	−	60	0	MT	125 mg; 3/day	DP and IT	50
Shao L [10]	Retrospective	Single center	−	149	0	FL and MT	125 mg; 3/day	DP and IT	50
Qin N [18]	Retrospective	Single center	DNA sequencing	101	0	FL and MT	125 mg; 3/day	DP and IT	50
Zheng X [23]	Retrospective	Single center	Direct sequencing	42	0	FL	125 mg; 3/day	DP and IT	52
Hu X [12]	Prospective	Multiple center	−	−	−	−	125 mg; 3/day	DP and IT	53
Pan H [17]	Retrospective	Single center	−	69	0	FL and MT	125 mg; 3/day	DP and IT	51
Chen X [8]	Retrospective	Single center	−	82	0	FL and MT	125 mg; 3/day	DP and IT	50
Li X [15]	Retrospective	Single center	ARMS−PCR	124	0	FL and MT	125 mg; 3/day	DP and IT	48
Hu X [13]	Retrospective	Multiple center	−	124	4	FL and MT	125 mg; 3/day	DP and IT	50
Wang T [21]	Retrospective	Single center	−	67	0	−	125 mg; 3/day	DP and IT	52
Shen YW [19]	Retrospective	Single center	ARMS−PCR	35	0	−	125 mg; 3/day	DP and IT	49
Jiang X [14]	Retrospective	Single center	−	231	0	FL and MT	125 mg; 3/day	DP and IT	47

Abbreviations: FL, first line therapy of icotinib; MT, maintenance treatment (the second line or above); TREND, the transparent reporting of evaluations with nonrandomized designs; DP, disease progression; IT, intolerable toxicity; ARMS-PCR, amplification refractory mutation system: MELA, mutant-enriched liquid array.

### Quality and heterogeneity of literature

Most of 15 studies [8, 11, 12, 14–24] were retrospective, non-randomized, parallel, controlled study (observational study). Because the research and effect evaluation of clinical new drug involves the ethical and moral risk, therefore most of studies included in this analysis are non-randomized current controlled trials (NRCCT). The random assignment of cases was somewhat unsatisfactory, but there are relatively few other methodological problems (Figure 1B and Figure 1C) in these studies. In addition, TREND statement showed that the scores of eleven studies were more than 50 [8, 10, 12, 13, 16–18, 20–23], the rest of the four studies showed to be between 47 to 49 [11, 14, 15, 19]. In terms of heterogeneity, the Chi-squarevalues of heterogeneity test on comparison of ORR and DCR were 6.88 and 9.11 with 10 degree of freedom (d.f.) and *p* = 0.737 and 0.522 respectively, and the I^2^ statistical values were 0% respectively. Together, the heterogeneity did not exist in these studies, so we used a method of fixed models to combine all items.

### Efficacy evaluation of icotinib in treating NSCLC

Fifteen studies [8, 11, 12, 14–24] reported the ORR of icotinib in treating NSCLC and the pooled ORR was 40.99% (95% CI: 33.77% to 48.22%). Thirteen of 15 studies [8, 11, 12, 15–20, 22–24] offered the data of DCR and the pooled DCR was 77.16% (95% CI: 51.43% to 82.31%). The data on PFS was given by 11 studies [8, 14–20, 23, 24] and the pooled mean PFS was 7.34 months (95% CI: 5.60 to 9.07). Five of 15 studies [8, 10, 13, 19, 23] provided the data on OS and that was 14.98 months (95% CI: 9.78 to 20.18).

### Efficacy comparison of icotinib in treating NSCLC patients between EGFR mutations and EGFR wild type gene

**Figure 2:**
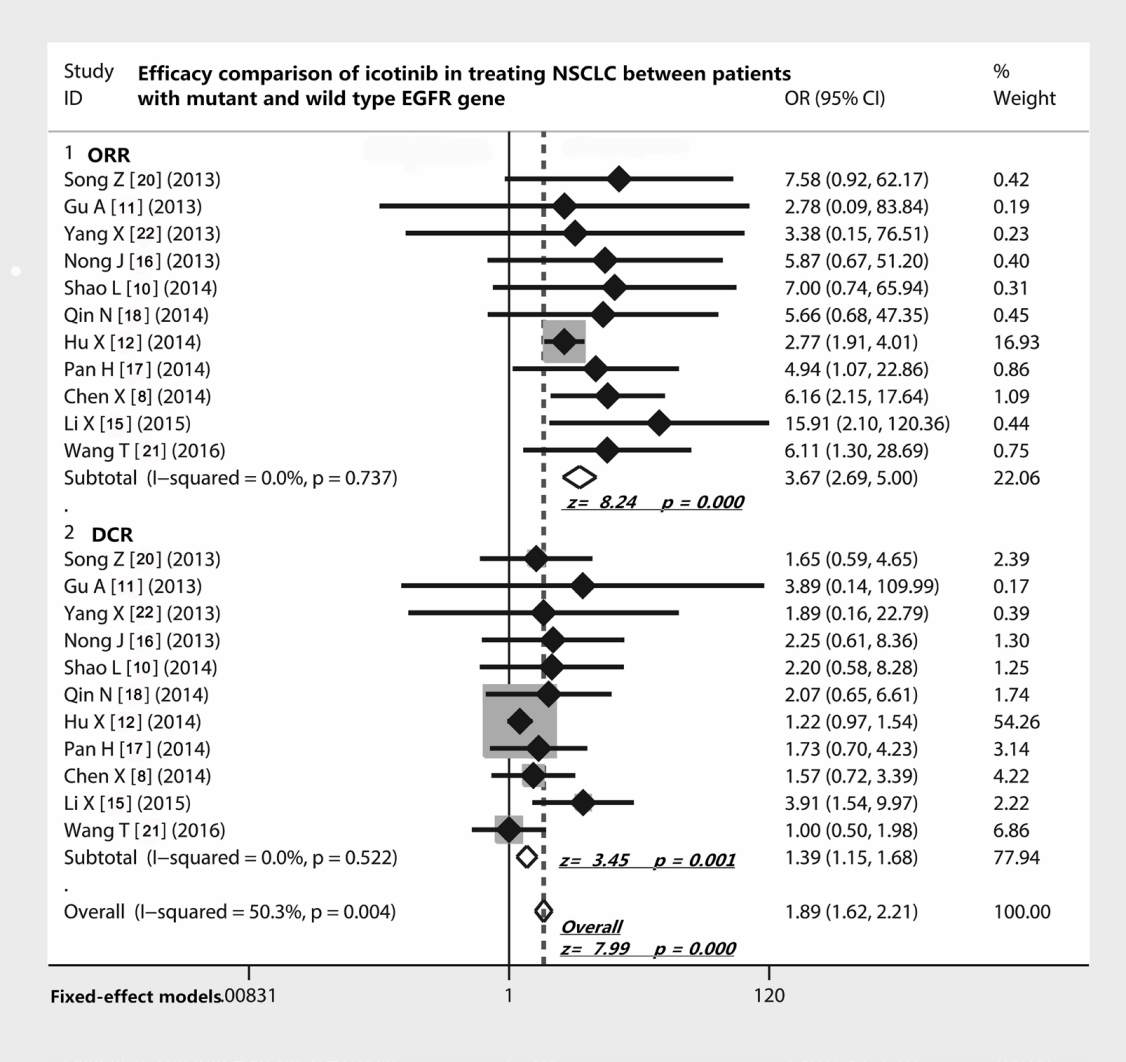
Efficacy comparison of icotinib in treating NSCLC patients with EGFR mutations and EGFR wild type gene Patients with EGFR mutants had a remarkably higher ORR to icotinib in treating NSCLC; patients with EGFR mutants also showed a relatively higher DCR compared with those with wild type EGFR gene. Abbreviations: EGFR, epithelial growth factor receptor; NSCLC, non-small cell lung cancer; ORR, overall response rate; DCR, disease control rate; OR, odds ratio.

**Figure 3:**
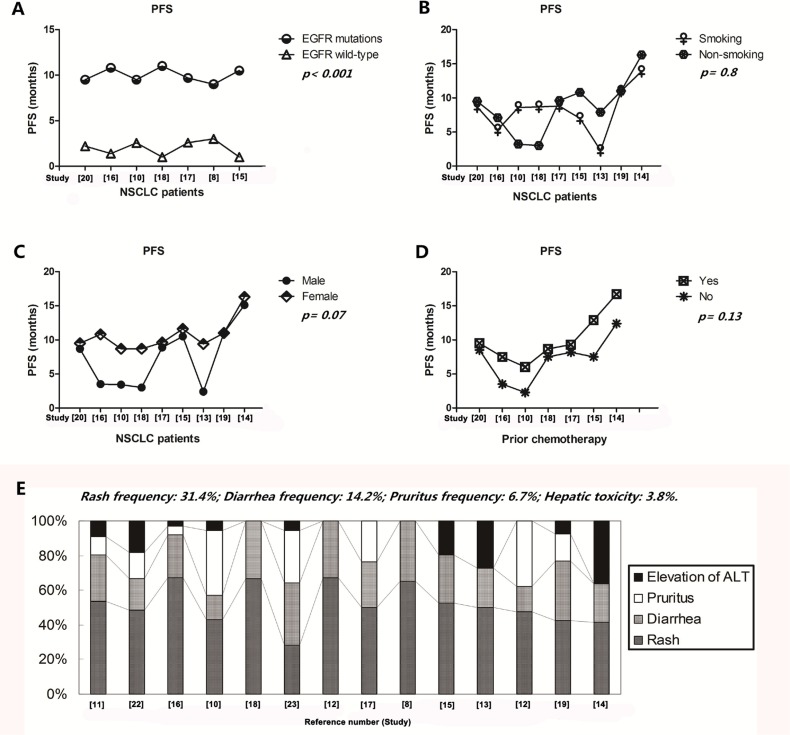
Influence of icotinib on PFS and tolerability in treating NSCLC patients with different clinical parameters (**A**) Patients with EGFR mutants also showed a relatively longer PFS compared with those with wild type EGFR gene; (**B**–**D**) smoking, gender and prior chemotherapy did not have influences on the PFS when they received the therapy of icotinib; (**E**) the most common AEs were rash, diarrhea, pruritus and hepatic toxicity when patients accepted the icotinib therapy; Abbreviations: PFS, progression free survival; EGFR, epithelial growth factor receptor; NSCLC, non-small cell lung cancer; ALT, alanine transaminase.

As shown in Table 3, eleven studies [8, 11, 12, 15–18, 20–22, 24] reported the difference of ORR and DCR of icotinib in treating NSCLC between patients with EGFR mutations and wild type EGFR gene. The odds ratio of ORR was 3.67 (95% CI 2.69 to 5; Z = 8.24, *p* < 0.001) and of DCR was 1.39 (95% CI 1.15 to 1.68; Z = 3.45, *p* = 0.001), which indicated that patients with EGFR mutations had better ORR and DCR to the treatment of icotinib compared with those without mutations (Figure 2). In addition, as seen in Figure 3A, patients with EGFR mutations showed a longer PFS than those without mutations (11.0 ± 0.76 months vs. 1.97 ± 0.82 months) (t = 18.94, df = 12, *p* < 0.001).

**Table 3:** The efficacy of icotinib in EGFR mutation and wild-type patients

Author	Assessment of overall efficacy				Assessment of efficacy regarding the status of EGFR mutations										PFS (months)	
	Overall efficacy				Control design (N)		EGFR mutations (N)				EGFR wild-type (N)				EGFR mutation	EGFR wild-type
	ORR (%)	DCR (%)	PFS (months)	OS (months)	EGFR Mutations	EGFR wild-type	CR	PR	SD	PD	CR	PR	SD	PD		
Song Z [20]	44.8	79.6	8.5	−	36	13	0	21	11	4	0	1	6	6	9.5	2.2
Gu A [11]	36	69.7	−	−	4	2	0	2	1	1	0	0	0	2	−	−
Yang X [22]	46.4	78.6	−	−	18	2	0	12	5	1	0	0	1	1	−	−
Nong J [16]	45	80	6.7	−	23	9	15		8	0	1		3	5	10.8	1.4
Shao L [10]	22.1	71.8	5.03	12.3	12	12	7		4	1	1		4	7	9.5	2.57
Qin N [18]	37.6	79.2	6.5		35	11	18		15		1		5		11	1
Zheng X [23]	33.3	85.7	7	13	2	6	−		−	−	−		−	−	−	−
Hu X [12]	30.3	80.6	−	−	665	214	327		287		38		124		−	−
Pan H [17]	43.5	76.8	8.6	−	51	18	0	28	16	7	0	2	7	9	9.7	2.6
Chen X [8]	41	65	4	11	19	63	1	11	5	17	0	7	29	36	9	3
Li X [15]	51.6	79.8	6.0	−	99	25	0	63	30	6	0	1	5	19	10.5	1.0
Hu X [13]	25.8	67.7	5	17.6	−	−	−	−	−	−	−	−	−	−	−	−
Wang T [21]	26.7	−	−	−	38	29	0	16	22	0	0	2	27	0	−	−
Shen YW [19]	62.9	88.6	11	21	35	0	−	−	−	−	−	−	−	−	−	−
Jiang X [14]	67.9	−	12.4	−	−	−	−	−	−	−	−	−	−	−	−	−

Abbreviations: N, cases; ORR, overall response rate; DCR, disease control rate; PFS, progression-free survival; OS, overall survival; EGFR, epidermal growth factor receptor; CR, complete response; PR, partial response; SD, stable disease; PD, progressive disease.

### Influence of icotinib on PFS in treating NSCLC patients with different clinical parameters

As shown in Table 4, nine studies [13–20, 22, 24] provided the data on the correlation between PFS of icotinib and smoking or gender, which showed that the status of smoking (*t* = 0.26, df = 16, *p* = 0.8) and gender of patients (*t* = 1.91, df = 16, *p* = 0.07) did not have influences on the PFS when they received icotinib (Figure 3B and Figure 3C). Seven studies [14–18, 20, 22, 24] concerning the relationship between the PFS and prior chemotherapy showed that the PFS of first line group (10.1 months) and the second line (7.1 months) did not have a statistical difference when they received icotinib (*t* = 1.59, df = 12, *p* = 0.13) (Figure 3D).

**Table 4:** Univariate analysis of included studies

Author	PFS						Adverse events (all)				Adverse events							
	Smoking		Gender		Prior chemotherapy						Rash				Diarrhea			
	Yes	No	Male	Female	0	≥1	Rash (%)	Diarrhea (%)	Pruritus (%)	Elevation of ALT %)	ORR (Yes)	ORR (No)	DCR (Yes)	DCR (No)	ORR (Yes)	ORR (No)	DCR (Yes)	DCR (No)
Song Z [20]	8.7	9.5	8.7	9.5	9.5	8.5	−	−	−	−	−	−	−	−	−	−	−	−
Gu A [11]	−	−	−	−	−	−	33.7	16.9	6.7	5.6	−	−	−	−	−	−	−	−
Yang X [22]	−	−	−	−	−	−	28.6	10.7	8.9	10.7	11/16	15/40	16/16	28/40	1/6	1/25	25/50	38/50
Nong J [16]	5.3	7.1	3.5	10.8	7.5	3.5	38.3	14.2	2.8	1.7	−	−	−	−	−	−	−	−
Shao L [10]	8.6	3.23	3.43	8.7	6.03	2.27	15	5	13	2	14/37	19/112	30/37	77/112	−	−	−	−
Qin N [18]	8.7	3	3	8.7	8.7	7.5	35.6	17.8	−	−	21/36	17/65	35/36	45/65	8/18	30/83	17/18	63/83
Zheng X [23]	−	−	−	−	−	−	35.7	45.2	38.1	7.1	−	−	−	−	−	−	−	−
Hu X [12]	−	−	−	−	−	−	17.4	8.5	−	−	−	−	−	−	−	−	−	−
Pan H [17]	8.8	9.6	8.9	9.6	9.3	8.2	24.6	13	11.6	−	−	−	−	−	−	−	−	−
Chen X [8]	−	−	−	−	−	−	39	20.7	−	−	−	−	−	−	−	−	−	−
Li X [15]	7	10.8	10.5	11.6	12.9	7.5	30.6	16.1	−	11.3	−	−	−	−	−	−	−	−
Hu X [13]	2.3	7.9	2.4	9.4	−	−	26.6	12.1	−	14.5	−	−	−	−	−	−	−	−
Wang T [21]	−	−	−	−	−	−	43.3	13.4	34.3	−	11/38	5/38	−	−	−	−	−	−
Shen YW [19]	11	11	11	11	−	−	31.4	25.7	11.4	5.7	−	−	−	−	−	−	−	−
Jiang X[14]	13.9	16.3	15.1	16.3	16.7	12.4	22.1	11.7	−	19.5	−	−	−	−	−	−	−	−

Abbreviations: N, cases; ORR, overall response rate; DCR, disease control rate; PFS, progression-free survival.

### Tolerability of icotinib maintenance therapy

As shown in Figure 3E, the most common AEs of icotinib therapy were rash [31.4 (31.4 ± 8.26)%; 95% CI, 26.1–35.3], diarrhea [14.2 (14.2 ± 9.5)%; 95% CI, 11.7–22.3], pruritus (6.7%; 95% CI, 2.07–14.5) and hepatic toxicity [(3.8 (3.8 ± 6.3)%]. Most of them were 1–2 grade and could be well tolerated.

### Relationship between the efficacy of icotinib and the occurrence of rash and diarrhea

**Figure 4:**
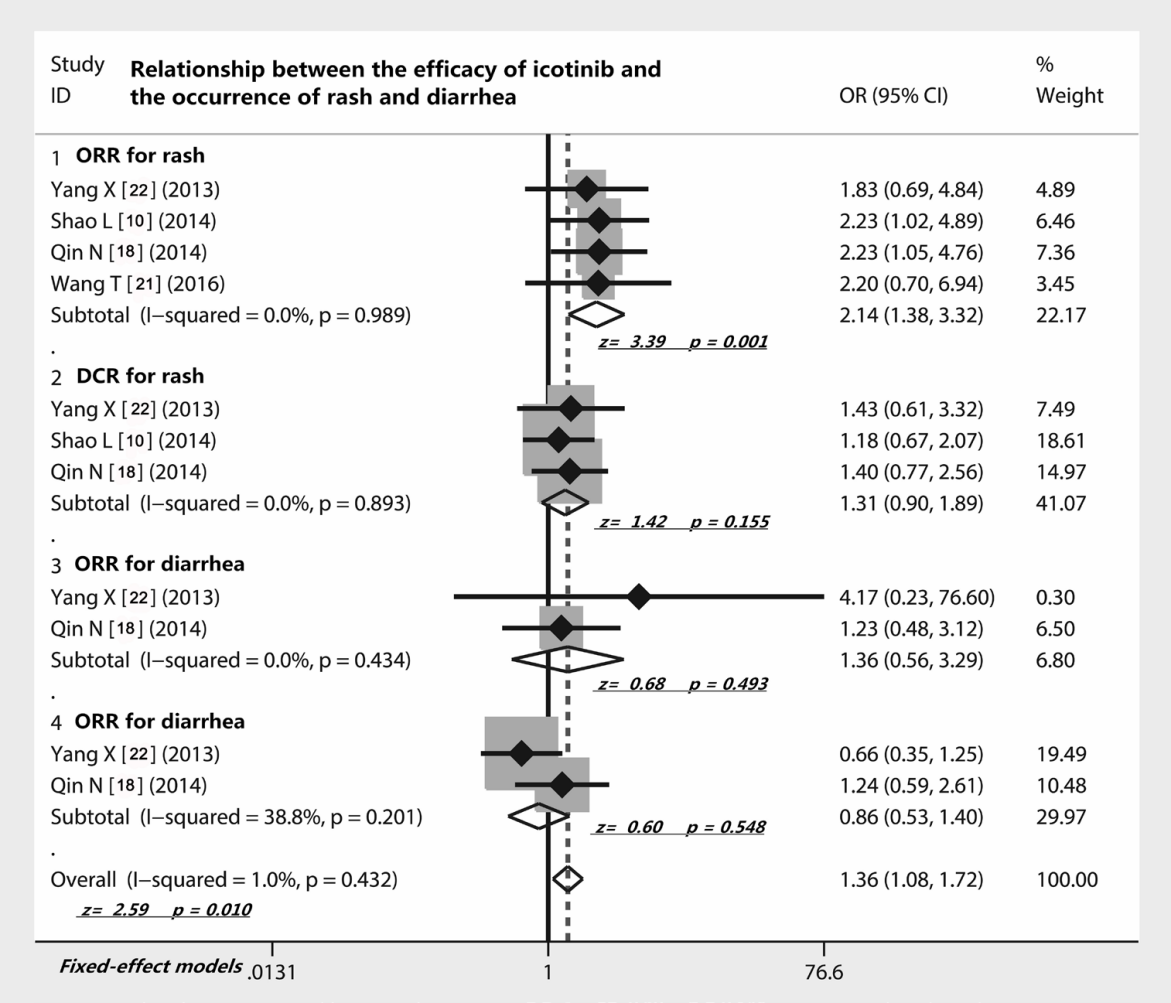
Relationship between the efficacy of icotinib and the occurrence of rash and diarrhea The presence of rash of NSCLC patients correlated with the ORR of icotinib but not DCR; the presence of diarrhea of NSCLC patients did not correlate with the ORR and DCR of icotinib; Abbreviations: ORR, overall response rate; DCR, disease control rate; OR, odds ratio.

Four studies [10, 18, 21, 22, 25] investigated the relationship between efficacy of icotinib and occurrence of rash, which showed that the ORR of icotinib in patients with rash was significantly higher than those who did not have rash (OR = 2.14; 95% CI 1.38 to 3.32; Z = 3.39, *p* = 0.001) (Figure 4). However, the analysis of three studies [10, 18, 22] exhibited that the DCR of icotinib was not related to the occurrence of rash (OR = 1.31, 95% CI 0.90–1.89; Z = 1.42; *p* = 0.155) (Figure 4). In addition, two studies [18, 22] compared the efficacy of icotinib and the occurrence of diarrhea. We found that either ORR (OR = 1.36; z = 0.68, *p* = 0.493) or DCR (OR = 0.86; z = 0.60, *p* = 0.548) of icotinib therapy all did not correlate with the incidence of diarrhea (Figure 4).

### Analysis of sensitivity and publication bias

**Figure 5:**
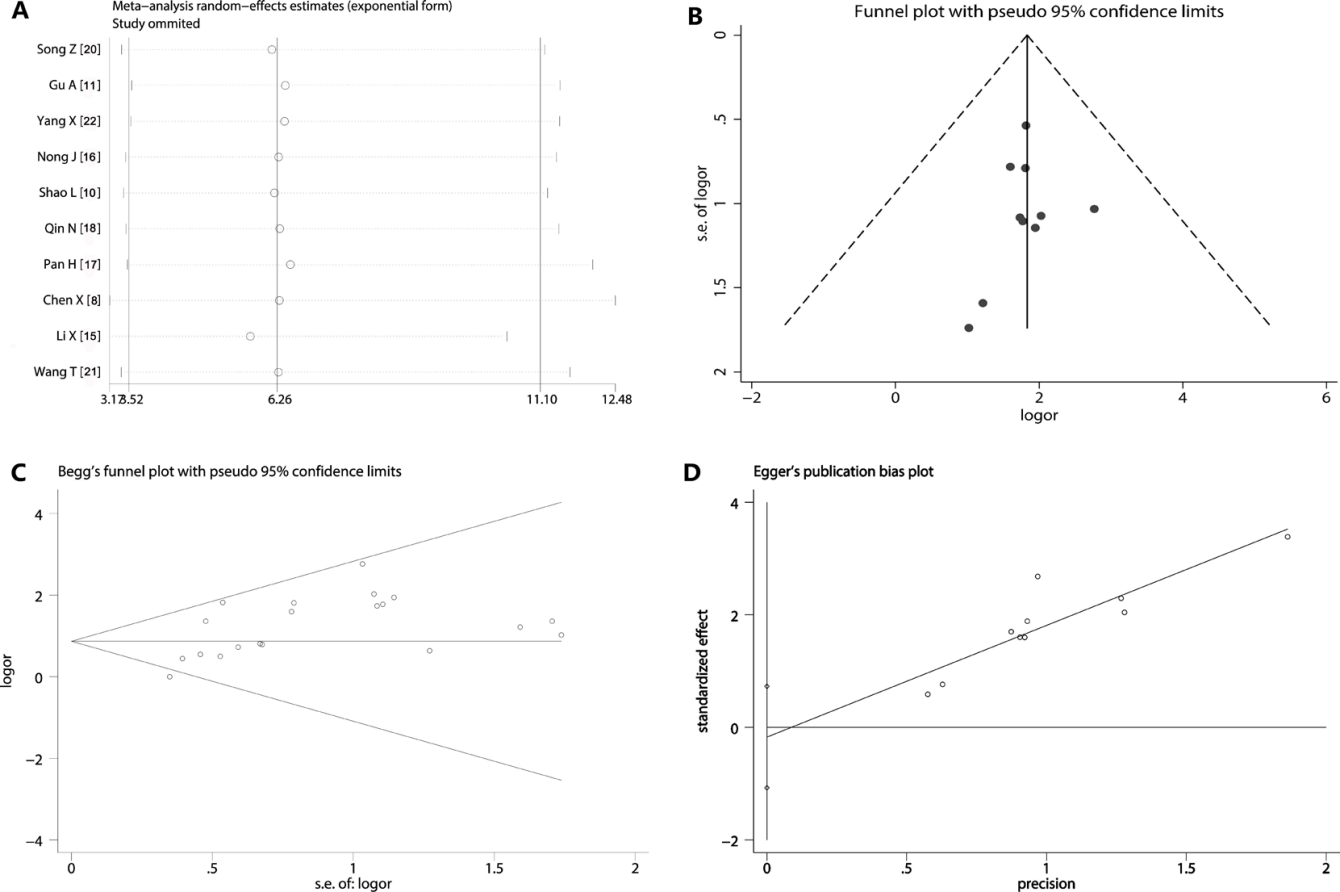
Sensitivity analysis and publication bias (**A**) The exclusion of studies individually did not substantially modify the estimators, with OR values varying between 2.77 and 15.91; (**B**) the shape of the funnel appeared to be approximately symmetrical; (**C**) Begg's test (*p* = 0.283) suggested that there was not publication biases; (**D**) Egger's test (*p* = 0.67) indicated that publication biases did not exist.

Sensitivity analysis showed that removing any one study did not exert a substantial impact on the overall effect value of this meta- analysis. The weight value of those studies vacillated from 2.77 to 15.91. Even if one of them had a large population of patients [12], removing it from the included studies did not change the overall effect yet (Figure 5A). In addition, the funnel plot of this meta-analysis seemed to be symmetrical (Figure 5B). And the Begg's test (SD of score = 11.18, *p* = 0.283) (Figure 5C) and Egger's test (*t* = −0.44, *p* = 0.67) (Figure 5D) all suggested that there was not a possibility of publication biases that would influence the stability of the results.

## DISCUSSION

EGFR-TKIs have been researched and developed as effective targeted anti-tumor drugs for NSCLC [4]. The representatives of them, gefitinib and erlotinib, have displayed an encouraging clinical efficacy [3]. Icotinib hydrochloride, a new product of EGFR-TKIs, was researched and developed by Betta Pharmaceuticals Co., Ltd, Hangzhou, China [7]. In 2011, it was approved by the China Food and Drugs Administration (CFDA) for treating advanced NSCLC [7]. As to a synthetic anilinoquinazoline compound like gefitinib and erlotinib, it can impede the cascade signaling of EGFR pathway by competitively binding to the EGFR-tyrosine kinase domain, blocking subsequent receptor autophosphorylation and downstream signaling [7]. During the past five years, much work has been done to investigate the efficacy and safety of icotinib in treating NSCLC. However, not all studies had the same conclusion; in order to assess this new drug, we did this systematic evaluation. To be clear, quality assessment of included studies is very important to meta-analysis. We used the criteria made by the Cochrane Handbook (Version 5.0.1) and the TREND statement to assess the quality of included studies and found that they had relatively few methodological problems and very good clinical homogeneity. In addition, a series of statistical analysis showed that there had not been an existence of heterogeneity. Thus, these studies can provide us reliable and useful evidence.

Previous studies have showed that the first-line and maintenance therapy of gefitinib and erlotinib had an encouraging clinical efficacy in treating NSCLC patients [26, 27]. Larger sample study also shows that the first-line or maintenance therapy of gefitinib significantly promotes the ORR, prolongs PFS, and improves the health-related quality of life [3]. One meta-analysis shows that erlotinib could benefit patients with EGFR mutation in terms of PFS [28]. We included 15 studies with a total of 2,304 individuals that concerned the efficacy and safety of icotinib as a first-line or maintenance therapy in NSCLC. Encouragingly, the ORR and DCR of icotinib respectively reached to 40.99% and 77.16%, which were higher than 18.4% and 54.4% in the IDEAL-1 study, and 11.8% and 42.0% in the IDEAL-2 study [29, 30]. Actually, after icotinib is approved by the CFDA in 2011, a large sample study included 6087 patients is performed as a single-arm, Phase IV study and shows that icotinib is an efficient and well-tolerated option for advanced NSCLC [6], which increases the confidence of using icotinib in China subsequently. After that, basic research demonstrates that icotinib has an almost similar structure of with erlotinib; however, the side-chain of icotinib has a feature of closed ring structure, which promotes its liposolubility and hydrophobicity. This structure feature makes icotinib easily pass through the cell membrane and thus increases its anti-tumor activity [3]. In our analysis, we also found that the PFS of icotinib therapy was 7.34 months and the OS was 14.98 months. ICOGEN study is a double-blind, phase 3 non-inferiority trial which enrolled patients with advanced non-small-cell lung cancer from 27 sites in China, which shows that icotinib has the same efficacy with gefitinib in terms of PFS (HR 0.84, 95% CI 0.67–1.05; median PFS 4.6 vs 3.4 months; *p* = 0.13) [31].

As gefitinib and erlotinib, the status of EGFR mutations is also critical to the therapy of icotinib, because NSCLC with EGFR mutations has one distinct benefit over those without mutations [4]. NSCLC cell lines with EGFR mutations displays a superior suppression of icotinib (IC50 value is 0.67 nM), compared with those without EGFR mutations (IC50 value is 10 nM) [24, 32]. In our meta- analysis, we found that patients with EGFR mutations had a remarkably higher ORR (OR = 3.67) and DCR (OR = 1.39) to therapy of icotinib than those without EGFR mutations. In addition, we also observed that patients with EGFR mutations also had obvious benefit of PFS, compared with those without EGFR mutations (11.0 ± 0.76 months vs. 1.97 ± 0.82 months). Previously, the IPASS study documented prolonged PFS and better tumor response from gefitinib in EGFR-mutated patients [12]. And, the PFS of gefitinib and erlotinib in studies of BR.21 [33], INTREST [34], TITAN [35], and ISEL [36] range from 1 month to 4 months, and the OS range from 5.3 to 7.6 months. From our results, we found that whether first-line treatment or maintenance treatment, patients with EGFR mutations all had a obvious benefit of PFS, which indicated that icotinib regimen would be considerably advantageous for patients with the EGFR mutations. However, patients with the wild-type EGFR gene could also benefit to some extent, but obviously less than EGFR mutations. Further, we noticed that the status of smoking (*p* = 0.8), gender (*p* = 0.07) and prior chemotherapy (*p* = 0.13) of NSCLC patients did not have influences on the PFS when they received the therapy of icotinib. We considered that the main reason might be the relatively small cases of some studies and some studies seemed not to concern these clinical features.

In our investigation, all studies adopted the same treat project: oral 125 mg dose of icotinib, q8h/day. The most common AEs included skin rash (31.4%), diarrhea (14.2%), pruritus (6.7%) and hepatic toxicity (3.8%); most of which were grades 1 to 2 and were well tolerated. We identified these studies and found that patients older than 70 years also seemed to be well tolerable to icotinib treatment. The occurrence of AEs in our review was consistent with those observed in previous investigations on other tyrosine kinase inhibitors [4], while numerically less than those with gefitinib [37] and erlotinib [38]. What's surprising about the study is that the ORR of icotinib in patients with rash was significantly higher than those who did not have rash, which suggested that presence or absence of rash could predict efficacy of icotinib. Although rarely life-threatening, skin toxicity may cause significant physical and psycho-social discomfort. Research suggests that the presence and severity of skin rash is associated with improved clinical efficacy in patients receiving EGFR inhibitors [39]. A meta-analysis also asserts that skin rash and progression were found to be independent predictive factors for survival [40].

We should face that there are certain limitations in these studies of our review. First, most of studies are retrospective. Second, all of the subjects were Chinese. Third, the sample in some studies was relatively small. Four, some studies did not clear show the EGFR status of patients. Because the research and effect evaluation of clinical new drug involves the ethical and moral risk, therefore most of studies included in this analysis are non-randomized current controlled trials (NRCCT). Although these flaws, these studies still contain credible evidence pointing toward such new drug-- icotinib.

In summary, the results of this study illustrate icotinib is an effective and well tolerated regimen for Chinese patients with advanced NSCLC, especially among those with EGFR mutations. Further randomized trials with large population are required to provide more evidence for evaluating of icotinib in the treatment of NSCLC. In future, we should pay more attention to the studying on adjuvant therapy, maintenance therapy and combination of icotinib.

## MATERIALS AND METHODS

### Identification of studies

We searched the databases of Medline, Web of Science, Embase and Cochrane Library to collect relevant studies pertaining to the efficacy and safety of icotinib in treating NSCLC. The Medical Subject Term we used included ‘icotinib’, ‘BPI-2009H’, ‘icotinib hydrochloride’, ‘lung cancer’, ‘non-small cell lung cancer’, ‘lung adenocarcinoma’, and ‘NSCLC’. In addition, we also did a hand searching from the references of included literature and contacted with the authors of studies for the first-hand data if necessary.

### Criteria of inclusion and exclusion on studies

The inclusion criteria: (1) icotinib treatment as the first line and maintenance therapy; (2) fully published papers; (3) included at least one of the following outcomes (ORR, overall response rate; DCR, disease control rate; OS, overall survival; PFS, progression free survival; AEs, adverse effects); (4) investigations must have focused on EGFR mutations; and (5) medical ethics must be assured. The exclusion criteria: (1) non-first hand literature (reviews, meeting record, editorials, letters, and commumication); (2) rate of the defaulters in study was more than 20%; (3) patients in study received treatment with other chemotherapeutics simultaneously; and (4) repetition of the published data.

### Treatment project supervision of icotinib

Patients with NSCLC in included studies must have been confirmed by histology and cytology before receiving treatment of icotinib. The following two projects were considered meeting our research design: (1) patients were switched to receive the therapy of icotinib after received the first-line therapy of platinum-based doublet chemotherapy; and (2) patients with newly diagnosed NCLC directly received icotinib as the first-line. Administration methods: (1) patients received icotinib by oral medication and (2) 125 mg/each time, one time/eight hours. Termination condition of treatment: (1) disease progression (radiographic or obvious clinical) or (2) severe toxicity was observed.

### Data extraction of studies

The extracted items included authors, years of publication, study design, country and region, EGFR mutations, test method of mutations, follow-up, missing data, administration, and treatment responses. The treatment responses were considered as key data: complete response (CR), partial response (PR), stable disease (SD) or progressive disease (PD), OS, PFS and AEs.

### Quality assessment and heterogeneity test of studies

We mainly adopted the criteria of the Cochrane Handbook (Version 5.0.1) to assess the included studies, which was special for systematic reviews of interventions. We also adopted the transparent reporting of evaluations with nonrandomized designs (TREND) statement to measure the quality of studies, which includes 59 questions. If the answer to a question is true, then give a “√” [25]. And we implemented a sensitivity analysis to disclose whether single study may bias the analyses. As to the heterogeneity test, we employed the chi-square test and *I ^2^* value estimation to finish, and considered significant at *p* < 0.1 [41].

### Statistical analysis

We calculated the overall efficacy of icotinib using a method of descriptive statistics, including the mean of items, standard deviation and 95% CI (confidence interval). For the subgroup data, we employed the Fixed-effect and random-effect models of meta-analysis to combine the overall effects, which showed the pooled odds ratio (OR) and 95% confidence interval. In the absent of homogeneity, we used the fixed-effects models, or we used the random-effect models. We also compared other important clinical parameters such as EGFR mutations (yes *versus* no), smoking (yes *versus* no), gender (male *versus* female) and prior chemotherapy (no *versus* > 1). Further, we calculated the data of survival using Student's *T*-test, and One-WAY ANOVA Test. Moreover, we employed the Begg's and Egger's test to assess the publication bias of studies. The statistical software that we used in this study included SPSS (SPSS 19.0, Chicago, USA) and Stata version 13.0 (Stata Corporation, College Station, TX, USA). We defined that all *p* values were two-sided, and *p* < 0.05 as a statistical significance.

## Abbreviations

AEs: adverse effects
ARMS-PCR: amplification refractory mutation system
CFDA: China Food and Drug Administration
DCR: disease control rate
DNA: deoxyribonucleic acid
CI: confidence interval
EGFR: epidermal growth factor receptor
NSCLC: non-small cell lung cancer
LAC: lung adenocarcinoma
LSCC: lung squamous cell carcinoma
NRCCT: non-randomized current controlled trials
ORR: overall response rate
OS: overall survival
OR: odds ratio
PFS: progression-free survival
TREND: transparent reporting of evaluations with nonrandomized designs
TKIs: tyrosine kinase inhibitors
TNM: tumor node metastases
